# Whole-Genome Shotgun Assembly and Analysis of the Genome of *Shiraia* sp. Strain Slf14, a Novel Endophytic Fungus Producing Huperzine A and Hypocrellin A

**DOI:** 10.1128/genomeA.00011-14

**Published:** 2014-02-06

**Authors:** Huilin Yang, Ya Wang, Zhibin Zhang, Riming Yan, Du Zhu

**Affiliations:** a Key Lab of Protection and Utilization of Subtropic Plant Resources of Jiangxi Province, Jiangxi Normal University, Nanchang, Jiangxi, China; b Key Lab of Bioprocess Engineering of Jiangxi Province, Jiangxi Science and Technology Normal University, Nanchang, Jiangxi, China

## Abstract

Here, we report the draft genome sequence of *Shiraia* sp. strain Slf14 (China Center for Type Culture Collection [CCTCC] no. 209294), which is used to produce huperzine A and hypocrellin A. The genome sequence will allow for the characterization of the molecular mechanisms underlying its beneficial properties.

## GENOME ANNOUNCEMENT

Huperzine A (HupA), a lycopodium alkaloid isolated originally from *Huperzia serrata*, has attracted intense attention since its marked anticholinesterase activity was discovered by Chinese scientists (1). HupA has been marketed in China as a new drug for Alzheimer’s disease (AD) treatment and is currently used in the United States as a supplement for preventing further memory degeneration (2). However, the production of HupA from plants in large quantities is currently unsustainable because the plant resource is very scarce and the content of HupA in plants is extremely low (3). Hypocrellin A (HypA), a pigment isolated originally from the parasitic fungus *Hypocrella bambusae*, has a long history of use as a traditional medicinal agent to treat rheumatoid arthritis, gastric diseases, and skin diseases related to fungal infections (4). Surprisingly, HupA and HypA were recently found to be produced by various endophytic fungi, which are much more controllable than the plants due to simpler genetics and easier manipulation (3, 5). *Shiraia* sp. strain Slf14, which can produce huperzine A and hypocrellin A, is a novel endophytic fungus isolated by D. Zhu and his research team from *Huperzia serrata* in China (6). The fungus was collected in the China Center for Type Culture Collection (CCTCC) with the number 209294. Here, we report the first genome sequencing of *Shiraia* sp. Slf14 in an attempt to identify the HupA and HypA biosynthetic gene clusters, and sequencing of the genome may afford a basis for subsequent research that is directly connected to the field of natural product synthesis.

The genome was sequenced using the Illumina Solexa HiSeq 2000 instrument at the Beijing Genomics Institute (BGI), Shenzhen, China. A library containing 500-bp inserts was constructed. Sequencing was performed with the paired-end strategy of 90- and 90-bp reads to produce 2.0 Gb of filtered sequences, representing 62.5-fold coverage of the genome. The sequences were assembled into 288 contigs (>1,000 bp) using the SOAP*denovo* software (7). The *Shiraia* sp. Slf14 chromosome is about 32 Mbp in length, with an average G+C content of 47.95%. Augustus was used as predictors. A total of 12,513 proteins were compared against the NCBI nonredundant (NR) protein databases by BLAST to forecast their biological functions (8).

The genome sequence of *Shiraia* sp. Slf14 is a valuable resource for identifying the genes involved in the biosynthesis of HupA and HypA, and it also serves as a platform for facilitating comparative genomics with other *Shiraia* sp. fungi. A putative HupA biosynthetic gene cluster is found in the *Shiraia* sp. Slf14 genome, and the cluster has been deposited in the Genbank database (accession no. KF915812). Gene expression analysis and bioassays are needed for further investigation into these genes. The genome sequence will accelerate the progress of research on *Shiraia* sp. Slf14.

### Nucleotide sequence accession numbers.

This whole-genome shotgun project has been deposited at DDBJ/EMBL/GenBank under the accession no. AXZN00000000. The version described in this paper is version AXZN01000000.
